# Proteomic profiling of olfactory exfoliates from people with subjective cognitive complaints reveal networks of olfactory biomarkers of cognitive performance

**DOI:** 10.3389/fnagi.2026.1781518

**Published:** 2026-05-21

**Authors:** Tamal Sadhukhan, Narayan Rai, Maria Mañanita S. Hipolito, Myeshia Shelby, Claudia Ivonne Mejía Mondragón, Sriparna Sadhukhan, Abimbola Idowu, Rency S. Varghese, Muhammad Salman Sajid, Habtom W. Ressom, Adedoyin Kalejaiye, Thomas O. Obisesan, Magdalena Misiak-Christian, Evaristus A. Nwulia

**Affiliations:** 1 Department of Psychiatry and Behavioral Sciences, Howard University, Washington, DC, United States; 2 Lombardi Comprehensive Cancer Center, Department of Oncology, Georgetown University Medical Center, Washington, DC, United States; 3 Department of Oncology, Genomics and Epigenomics Shared Resources, Georgetown University, Washington, DC, United States; 4 Division of Otolaryngology/Head and Neck Surgery, Department of Surgery, Howard University, Washington, DC, United States; 5 Department of Internal Medicine, Howard University, Washington, DC, United States; 6 Department of Physiology and Biophysics, Howard University, Washington, DC, United States

**Keywords:** Alzheimer’s disease, ingenuity pathway analysis, liquid chromatography–tandem mass spectrometry, logical memory II recognition, mild cognitive impairment, olfactory mucosa, Wechsler Memory scale

## Abstract

**Introduction:**

Partly due to the inaccessibility of olfactory brain regions vulnerable to early Alzheimer’s Disease (AD) for repeated sampling, proteomic networks underlying progressive cognitive decline remain poorly understood. The olfactory mucosa (OM), an accessible part of the olfactory system, reflects central nervous system physiology and pathology, and represents a promising site for biomarker discovery. This study aimed to identify olfactory proteomic markers and pathways associated with performance in the logical memory II recognition (LM II_recog) subtest of the Wechsler Memory Scale among older adults with subjective cognitive complaints.

**Methods:**

Clinical, olfactory, and cognitive assessments were conducted on 108 adults aged 55–85 years from the Washington, DC region. Nasal exfoliates were sampled from the upper nasal cavities, and protein extracts from these samples were analyzed by mass spectrometry (MS). Linear regression with false discovery rate (FDR) correction (*q* < 0.1) was used to identify proteins associated with LM II_recog performance, and ingenuity pathway analysis (IPA) was applied to determine functional pathways.

**Results:**

A total of 137 proteins meeting the FDR *q* < 0.1 threshold were found to be linearly correlated with LM II_recog scores. Of the top 10 most significant proteins, six (PLOD1, MFN2, NGFR, PPP2R5E, C4A/C4B, and ITGAV) have previously been linked to AD and/or cognitive function, underscoring their potential as biomarkers of cognitive impairment. Ingenuity pathway analysis using the knowledge base machine learning (ML) platform revealed several disease pathways highly represented among the significant proteins. These included Hyperactive Behavior, Neuromuscular Disease, Tauopathy, Behavioral Deficits, Alzheimer’s Disease, Progressive Dementia, Degenerative Dementia, Alzheimer’s or Frontotemporal Dementia, all of which were associated with LM II_recog performance in the elderly population.

**Discussion:**

This study demonstrates the feasibility of using OM-derived proteomics to identify molecular signatures associated with cognitive performance and highlights the OM as a potential site for non-invasive biomarker discovery. These findings provide a foundation for future studies integrating OM profiling with established AD biomarkers.

## 1 Introduction

As people age, memory and other cognitive domains also diminish, but not all age-related cognitive decline is resultant of, or leads to, Alzheimer’s Disease and Related Dementias (ADRD). A better understanding of molecular mechanisms of age-related cognitive decline in the nervous system is important for elucidating the pathophysiology of normal and pathological cognitive changes in older populations. The repeated sampling of brain tissues from individuals with cognitive complaints would provide the most direct insight into the molecular pathophysiology of ADRD risk, however, such an approach is not ethically or practically feasible in living individuals, necessitating the use of accessible surrogate tissues. Therefore, alternative biomolecular sources from nervous system tissues that reflect early pathophysiology, such as the olfactory system, are needed to study molecular mechanisms of cognitive measures that are sensitive to preclinical changes in ADRD.

Memory is a multifaceted construct comprising interconnected processes of encoding, storage, and retrieval (Richter-Levin and Akirav, 2003). The LM subtest of the Wechsler Memory Scale – Fourth Edition (WMS-IV) is widely recognized as a robust clinical tool for assessing verbal episodic memory, encompassing the processes of encoding, storage, and retrieval (Ahn et al., 2020; Sullivan et al., 2000). The LM subtest reflects real-world memory demands—recalling conversations, events, and personal experiences—essential for daily functioning. It integrates multiple cognitive processes (Ahn et al., 2020; Sullivan et al., 2000) and distinguishes normal aging, mild cognitive impairment (MCI), and Alzheimer’s disease, while predicting conversion to AD dementia (Li et al., 2006; Rabin et al., 2009) LM performance depends on hippocampal and medial temporal lobe integrity—regions affected early in Alzheimer’s disease—and may detect subtle cognitive changes years before clinical diagnosis, making it a sensitive marker of early decline (Sperling et al., 2011). Despite its clinical utility, the biological underpinnings of LM performance remain underexplored, particularly the proteomic signatures that may correlate with LM II_recog outcomes. This knowledge gap is especially pronounced in African American populations, who are disproportionately affected by AD yet remain underrepresented in biomarker research. Identifying such proteomic correlations could provide novel insights into the molecular basis of memory function and serve as a foundation for biomarker development in early dementia detection.

The olfactory mucosa (OM) contains olfactory receptor neurons whose axons project directly to the olfactory bulb, along with sustentacular and basal cells (Mori and Sakano, 2011; Schwob, 2002; D'Anniballe et al., 2026). Nasal swab-derived exfoliates from superior and middle nasal turbinates contain olfactory neuroepithelium, which consists of olfactory receptor neurons, supporting sustentacular cells, and neural stem cells, although it can contain shed epithelial cells as well. Basal neural stem cells can be induced to differentiate into neurons, providing a potential source for studying neurodegenerative diseases (Brozzetti et al., 2023; Lavoie et al., 2017). OM samples provide a biologically relevant proxy of the nasal–brain interface, which is implicated in early Alzheimer’s disease–related pathology (D'Anniballe et al., 2026; Misiak et al., 2017). APOE isoform–specific effects are evident early in life and are amplified with aging and metabolic stress (Filippini et al., 2009). In memory-related regions such as the hippocampus, APOE4 carriers often exhibit functional and structural alterations even in the absence of clinical cognitive impairment, suggesting that APOE genotype modulates baseline neuronal physiology as well as susceptibility to disease (Bookheimer et al., 2000). Collectively, these observations underscore the critical role of APOE isoform–dependent protein expression and function in hippocampal health and memory processes, providing a biological framework for understanding differential cognitive aging and neurodegenerative risk (Huang and Mahley, 2014). However, it is also noteworthy that the association between ε4 and Alzheimer’s Disease is very weak and inconsistent in populations of African descent (Murrell et al., 2006; Gureje et al., 2006).

A defining feature of the olfactory system is its lifelong, tightly integrated relationship with learning- and memory-related circuits, including limbic regions that are especially vulnerable in neurodegenerative disorders. Through direct connections to the entorhinal cortex, hippocampus, and amygdala, the olfactory system reflects early molecular and circuit-level changes that parallel preclinical AD pathology. Converging evidence indicates that olfactory dysfunction and circuit alterations precede overt cognitive decline and track neurodegenerative processes (Karalis and Sirota, 2022; Zhang et al., 2024; Zou et al., 2016), highlighting the importance of investigating olfactory circuitry in aging. Logical memory is closely tied to hippocampal function (Petersen et al., 2000). The olfactory mucosa, located in the nasal cavity, is structurally linked to the olfactory bulb (OB), which in turn connects indirectly to the hippocampus and entorhinal cortex through the primary olfactory cortex, specifically the piriform cortex (Nwulia et al., 2017; Sabaie et al., 2021). In a previous review publication (Misiak et al., 2017), we showed evidence from several studies that Alzheimer’s neuropathology is reflected across the entire olfactory system, from the OM to the transentorhinal and entorhinal cortices early in the preclinical phases (Arnold et al., 2010). Given its (Misiak et al., 2017) accessibility for repeated sampling, the OM provides a unique opportunity to probe hippocampus-related cognitive processes, such as logical memory, in living individuals—an approach not feasible with hippocampal biopsies.

The present pilot study investigated proteomic markers and functional pathways associated with LM II_recog performance in predominantly African American older adults residing in the Washington, DC area. Using an unbiased, untargeted tandem mass spectrometry (MS/MS) proteomic approach, we aimed to identify protein signatures linked to cognitive function and to uncover molecular pathways implicated in early cognitive impairment in this understudied population.

## 2 Materials and methods

### 2.1 Study subjects

A total of 108 adults aged 55–85 years were enrolled from the Howard University Geriatric Clinic and surrounding communities in the Washington, DC metropolitan area. The majority of participants (87%) self-identified as African American. This ongoing study, initiated in 2021, was approved by the Howard University Institutional Review Board (IRB) and conducted in accordance with the Declaration of Helsinki. All assessments were performed by trained research staff at Howard University. Participants were eligible if they were between 55 and 85 years of age, with no history of life-threatening medical conditions, stroke, intracranial disease or infection, human immunodeficiency virus (HIV)+, Alzheimer’s dementia, or current substance use disorders. Individuals with nasal obstruction, deformity, or severe acute nasal disease that could impair olfactory function, recent (less than 6 months prior to study) history of COVID-19, or autoimmune and chronic systemic inflammatory diseases were also excluded.

### 2.2 Cognitive and psychophysical olfactory measurements

All participants underwent a comprehensive cognitive assessment that included the Logical Memory (LM) II subtest of the Wechsler Memory Scale IV (WMS-IV) (Ahn et al., 2020). LM II Recognition was selected over delayed recall because it provides a more sensitive and specific measure of memory impairment in populations with variable encoding efficiency, minimizing confounding effects of attention and retrieval deficits that can influence delayed recall performance. The LM II_recog subtest of the WMS-IV specifically evaluates verbal episodic memory by requiring participants to respond “yes” or “no” to questions about a narrative they heard 20–30 min earlier (Ahn et al., 2020). In addition, the LM II Delayed Recall score (0–25 story units) was recorded to quantify recall performance. Other cognitive measures assessed include the Free and Cued Selective Reminding Test (FCSRT)(Teng et al., 2023), Mini-Mental Status Examination Score (MMSE) (Kistler-Fischbacher et al., 2025; Folstein et al., 1975) and the Digit Symbol Substitution Test (DSST) (Jaeger, 2018). Based on a previously published approach (Donohue et al., 2014), the Preclinical Alzheimer’s Cognitive Composite (PACC) was computed for each participant from the combination of four (4) tests, which are MMSE, FCSRT, DSST and Logical Memory II story b delayed recall.

Finally, psychophysical tasks of olfaction were conducted on all participants, using the Sniffin’ Sticks test (Hummel et al., 2007). This standardized test assesses three domains of olfaction – odor threshold, odor discrimination (OD), and odor identification (OI). Total scores from these domains were used to compute the threshold-discrimination-identification (TDI) composite score, which represents a validated measure of global olfactory performance for each participant (Hummel et al., 2007).

### 2.3 Nasal swab collection

As previously described, olfactory mucosa samples were collected from each participant using FLOQSwabs (Copan Italia, Brescia, Italy) by the otolaryngologist (AK) collaborator at Howard University Hospital (Bongianni et al., 2022). Swabs were obtained from both upper nasal cavities, which anatomically contains the highest density of olfactory cells, placed into 15-ml tubes, and immediately snap-frozen in liquid nitrogen. Samples were then stored at −80 °C until subsequent protein isolation. Before initiating the study design, we confirmed the presence of mature, differentiated olfactory neurons within the olfactory mucosal (OM) tissue. Fluorescence and differential interference contrast (DIC) microscopy were used to characterize cellular composition in cultured OM samples.

### 2.4 Blood DNA isolation and APOE genotyping

Blood samples were collected from each participant at the phlebotomy unit of Howard University Hospital (HUH). Genomic DNA was extracted from whole blood using the QIAamp Blood DNA Mini Kit (Qiagen, Cat. No. 51104) according to the manufacturer’s instructions. DNA purity and concentration were assessed with a NanoDrop spectrophotometer (Thermo Fisher Scientific, Waltham, MA, USA). For APOE genotyping, 2–20 ng of DNA was analyzed at SNPs *rs429358* and *rs7412* using TaqMan assays (Thermo Fisher Scientific; assay IDs C_3084793_20 and C_904973_10) with the TaqMan Fast Advanced Master Mix, following the manufacturer’s protocol (Zhong et al., 2016; Pehlivanoglu et al., 2024). Reactions were run on a QuantStudio 7 Real-Time PCR System (Applied Biosystems), and genotypes were determined using the QuantStudio Real-Time PCR Software algorithm (Thermo Fisher Scientific).

### 2.5 Proteomics sample preparation

Proteins were extracted from nasal swabs using Radio-Immunoprecipitation Assay (RIPA) buffer (Fisher Scientific, Cat# 89900) and quantified with the Bicinchoninic Acid (BCA) assay (Thermo Scientific, Cat# 23225) following the manufacturer’s protocol (Tsai et al., 2016; Tuli and Ressom, 2009). For digestion, 100 μg of proteins were dissolved in 7 M urea/100 mM ammonium bicarbonate. The protein solutions were transferred to spin filter units. After the centrifugation for 10 min at 14,000 × g, 150 μL of 50 mM Dithiothreitol (DTT) in 7 M urea/100 mM ammonium bicarbonate was added to each spin filter unit and incubated for 30 min at room temperature to denature and reduce. Spin filter units were centrifuged for 10 min at 14,000 × g to remove the DTT residues. Then, 160 μL of 55 mM Iodoacetamide (IAA) was added in 7 M urea/100 mM ammonium bicarbonate to each spin filter unit and again incubated for 30 min in dark. Spin filter units were centrifuged for 10 min at 14,000 × g to remove the IAA. After washing twice with 100 μL of 50 mM ammonium bicarbonate, and once with 10 mM ammonium bicarbonate, trypsin solution prepared in 10 mM of ammonium bicarbonate was added to the final protein to enzyme ratio is 1:30. Each spin filter unit was wrapped with parafilm and incubated at 37 °C for 16 h. Finally, collection tubes were replaced with new ones and centrifuged for 10 min at 14,000 × g to collect the peptides. Digested peptides were desalted using BioPureSPN Mini PROTO 300 C18 columns (The Nest Group, MA, USA). Columns were equilibrated with 100% acetonitrile (ACN) and conditioned with 1% trifluoroacetic acid (TFA). Bound peptides were washed with 1% TFA and eluted sequentially with 80 and 50% can with 0.1% TFA. Eluates were dried and reconstituted in 3% ACN/0.1% formic acid (FA) for quantification (NanoDrop, Thermo Scientific). HeLa digest (Thermo Fisher Scientific) was used as a system suitability control and was injected at regular intervals throughout the analytical run to monitor LC–MS/MS performance and stability.

### 2.6 Nano-LC–MS/MS analysis

MS analysis was conducted using an UltiMate 3,000 UHPLC system (Dionex/Thermo Scientific, San Jose, CA, USA) coupled to a Q-Exactive MS (Thermo Scientific, San Jose, CA, USA) equipped with a nanospray flex ion source (Tsai et al., 2016). 1 μg of peptides from each sample was injected after measuring with a nanodrop (Thermo Scientific, San Jose, CA, USA). For pre-concentration and cleanup, peptides were first passed through a trap column (C18 Acclaim PepMap, 75 μm × 20 mm, 100 Å) before separation on an analytical column (C18 Acclaim PepMap RSLC, 75 μm × 250 mm, 100 Å). 0.1% formic acid (FA) was mobile phase A and 80% ACN with 0.1% FA was mobile phase B. A multistage gradient of 145 min was applied at 35 °C. Initially, 4% of mobile phase B was maintained at a flow rate of 300 nL/min for 5 min, followed by a gradual increase to 35% over the next 120 min at a flow rate of 220 nL/min. Subsequently, mobile phase B increased to 95% over 5 min at 250 nL/min, then reduced to 4% at 300 nL/min, and maintained at this level until the end of the run. Using a nanospray flex ion source, peptides were directed toward the mass spectrometer at a voltage of 2.2 kV. The complete MS scan covered a range from 370 to 1850 m/z, with analytes detected in the Orbitrap at 70000 resolutions. For MS/MS fragmentation, the 10 most intense ions were chosen and processed in a high-energy collision dissociation (HCD) cell with normalized collision energy (NCE) of 27.5, operated at a resolution of 17,500.

### 2.7 Peptide annotation and LFQ

Peptide annotation and label-free quantification (LFQ) were performed in Proteome Discoverer 3.0 (Thermo Scientific, USA) using the CHIMERYS node and the UniProt human protein database (July 2023). The workflow included mass recalibration, Minora Feature Detector, spectrum selection, CHIMERYS, Percolator, and precursor detector nodes to address chimeric spectra. Search parameters were set with a precursor mass tolerance of 10 ppm, fragment mass tolerance of 0.02 Da, and trypsin specificity. Carbamidomethylation of cysteine (+57.021 Da) was set as a fixed modification, and methionine oxidation (+15.995 Da) as a variable. False discovery rate thresholds were 0.01 (high confidence) and 0.05 (medium confidence).

### 2.8 Bioinformatics and statistical analysis

Clustering within each sample group was assessed by principal component analysis (PCA) using LFQ intensities of all peptides to determine potential outliers. Proteins that were detected in less than 70% of each sample group were filtered out. The abundance data for all filtered peptides were log2-transformed, and missing values inherent to data-dependent acquisition (DDA) shotgun approaches were imputed using the k-nearest neighbors (KNN) feature-wise method. The data were then normalized by median to reduce variation between runs. Linear regression analysis was performed to assess the association between baseline normalized protein abundance levels and LM II_recog scores, adjusting for age, APOE4 carrier status, and sex as nuisance parameters. Sensitivity analyses were performed to assess the impact of educational attainment; however, as no significant associations were found between education and the top candidate proteins, it was excluded from the final regression models to maintain model parsimony. Robust standard errors from sandwich estimators, which are robust to non-normal distributions of outcome variables, were used to derive the *p* values for interpreting the statistical relationships between an analyte and the outcome (Huber, 1967; White, 1980). For multiple testing, FDR adjusted q-value of <0.1 was considered as significant (Benjamini et al., 2001). Network and pathway analyses were conducted using the Ingenuity Pathway Analysis software (IPA, QIAGEN Inc.), a web-based software, used for the analysis, integration, and interpretation of data derived from different omics experiments to get a hypothetical insight to find the candidate biomarkers and hence new targets for the particular study in the context of biological function (Davalieva et al., 2017).

## 3 Results

### 3.1 Demography and APOE genotypes of the study participants

A total of 108 participants (mean LM II_recog: 20.1) were recruited from the greater Washington, DC area. The cohort was predominantly female (70.4%) and African American (87%), with an age range of 55–83 years (mean 66.7 years) and mean education of 14.3 years (range 6–25 years). Demographic and clinical characteristics, including marital and employment status, are summarized in Table 1. APOE genotyping revealed that 43.9% of participants carried at least one APOE ε4 allele, while 3.7% were ε4/ε4 homozygotes. Genotype frequencies were as follows: ε3/ε3 (38.9%), ε3/ε4 (33.3%), ε2/ε3 (16.7%), ε2/ε4 (6.5%), and ε2/ε2 (0.9%).

**Table 1:** Demographic features and APOE genotype frequencies of study participants.

Demography	*N* = 108 (%)
Gender	
Male	32 (29.63)
Female	76 (70.37)
Race	
Asian	1 (0.93)
Biracial	1 (0.93)
Black	94 (87.04)
Other	1 (0.93)
white	11 (10.19)
Marital status	
Divorced	21 (19.44)
Married	25 (23.15)
Married twice	2 (1.85)
Never married	44 (40.74)
Separated	1 (0.93)
Widowed	15 (13.89)
Employment status	
Employed	17 (15.74)
Retired	57 (52.78)
Self employed	5 (4.63)
Unemployed	26 (24.07)
Unemployed (disability)	3 (2.78)
ApoE genotypes	
Non E4 carrier (E2/E2, E2/E3, E3/E3)	61 (56.48)
Heterozygous E4 (E2/E4, E3/E4)	43 (39.82)
E4 Homozygous (E4/E4)	4 (3.7)

Clinical characteristics of the study participants	Mean (SD)
Age	66.65 (7.71)
Education	14.29 (3.42)
MMSE	28.13 (2.053)
Story B	8.35 (4.11)
DSST	42.41 (15.93)
LM II_Recog	20.06 (3.56)
FCSRT	46.93 (1.96)

MMSE, mini mental state examination; DSST, digit-symbol-substitution test.

LM II, logical memory II; FCSRT, free and cued selective reminding test.

The mean MMSE score was 28.1 (range 21–30). The Alzheimer’s Disease Cooperative Study–Preclinical Alzheimer Cognitive Composite (ADCS-PACC) raw scores averaged 120.6 (range 28–184). The mean DSST score was 42.4 (range 9–96), FCSRT score averaged 46.93 (range 38–48), Logical Memory II story b Delayed Recall score ranges from 0–18 with average 8.35, LM II_recog ranged from 0–7, with a mean of 4.9. Correlational analysis (Table 2) showed significant positive correlations between LM II_recog and other cognitive measures: MMSE (*r* = 0.44), FCSRT (*r* = 0.28), ADCS-PACC (*r* = 0.65), Story B (*r* = 0.6642), TDI (*r* = 0.35) and DSST (r = 0.46). Also, comparing the 1:1 correlation between global olfactory function (TDI) to each of the 6 cognitive tasks, LM II_recog demonstrated the strongest correlation with TDI among all cognitive tests evaluated, thereby serving as the most appropriate “anchor” for a study focused on olfactory mucosal proteomics (Table 2). These findings support the use of LM II_recog as a sensitive measure of cognitive performance that is also correlated with olfaction, which is a strong predictor progression from subjective memory complaints to Alzheimer’s dementia. The mean ± SD of LM II_recog scores in ε4 carriers vs. non carriers were 20.0 ± 3.4 vs. 20.2 ± 3.7 (*p* = 0.80).

**Table 2:** Correlation matrix of clinical variables.

Measures	LM II_R	MMSE	FCSRT	DSST	Story B	ADCS-PACC	TDI
LM II_R	1.0000						
MMSE	0.4380***	1.0000					
FCSRT	0.2838**	0.2882**	1.0000				
DSST	0.4641***	0.3461**	0.1501	1.0000			
Story B	0.6642***	0.4962***	0.2689*	0.3995***	1.0000		
ADCS-PACC	0.5397***	0.4669***	0.2335*	0.7995***	0.6273***	1.0000	
TDI	0.3541***	0.2256*	0.1866	0.1238	0.1850	0.1417	1.000

***p < 0.001. **p < 0.01. *p < 0.05.

LM-R, logical memory II recognition; MMSE, mini-mental status examination; FCSRT, free and cued selective reminding test; ADCS-PACC, DSST, digit-symbol-substitution test; TDI, odor threshold, discrimination and identification index.

### 3.2 Identification of protein markers in regression analysis with LM II_recog score

**Figure 1:**
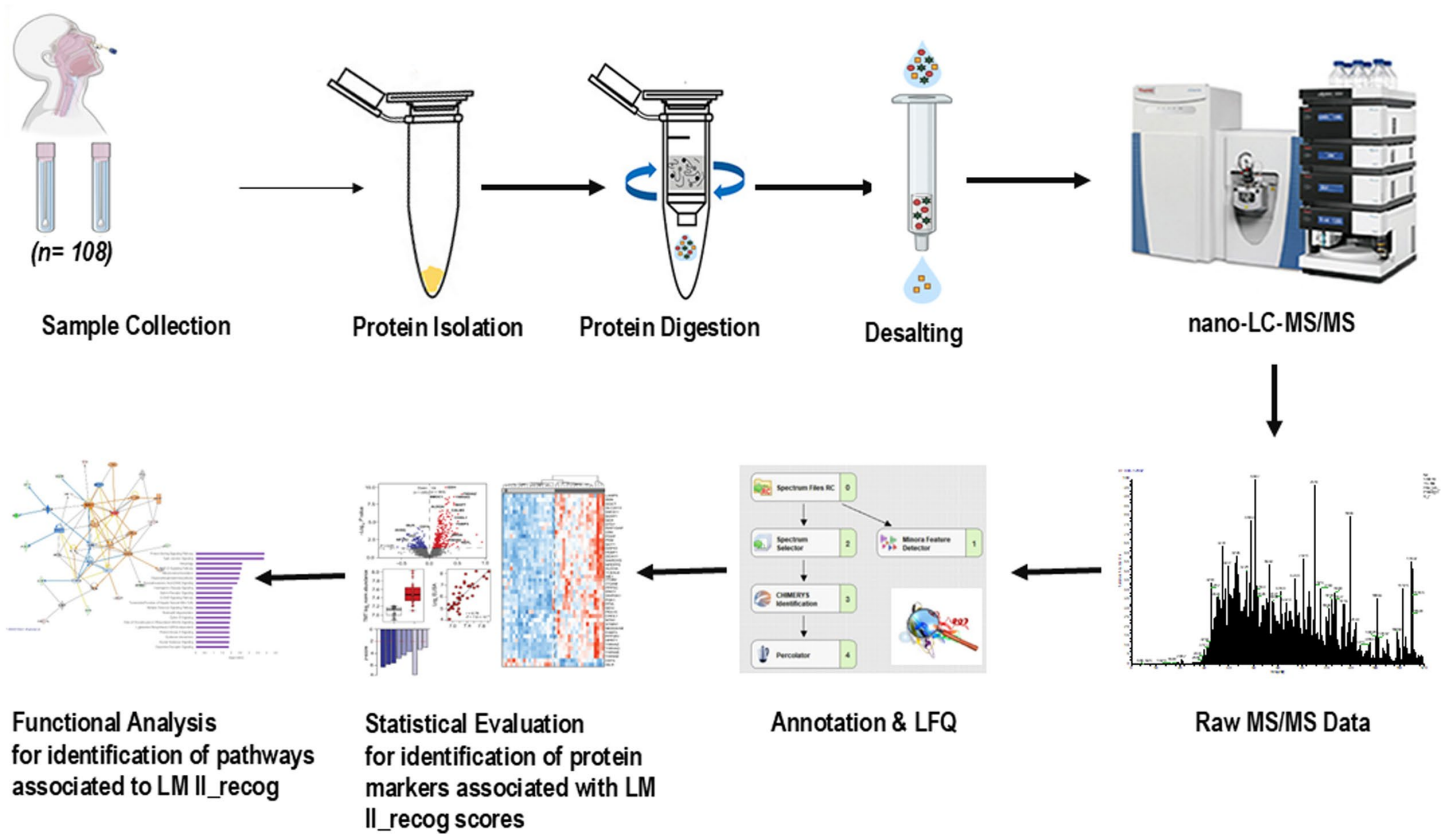
A schematic workflow of sampling and quantification of olfactory mucosa proteome.

Presence of olfactory cells in the nasal swabs was confirmed as depicted in the DIC images and neuronal and olfactory marker cell staining in the Supplementary Material (Supplementary Figures S1–S4). The workflow for the proteomic analysis of olfactory mucosa (OM) samples is summarized in Figure 1. OM swab samples were processed for protein extraction, enzymatic digestion, desalting, and analyzed by nanoLC–MS/MS on a Q-Exactive Orbitrap platform. The resulting MS/MS spectra were processed using Proteome Discoverer 3.0 with the CHIMERYS node, enabling high-confidence protein identification and quantification. To ensure robust data quality, quality control (QC) runs using HeLa protein digest standards were interspersed throughout the data acquisition process. The QC results confirmed consistent instrument performance, reproducibility of peptide detection, and high mass accuracy across the analytical batches.

**Figure 2:**
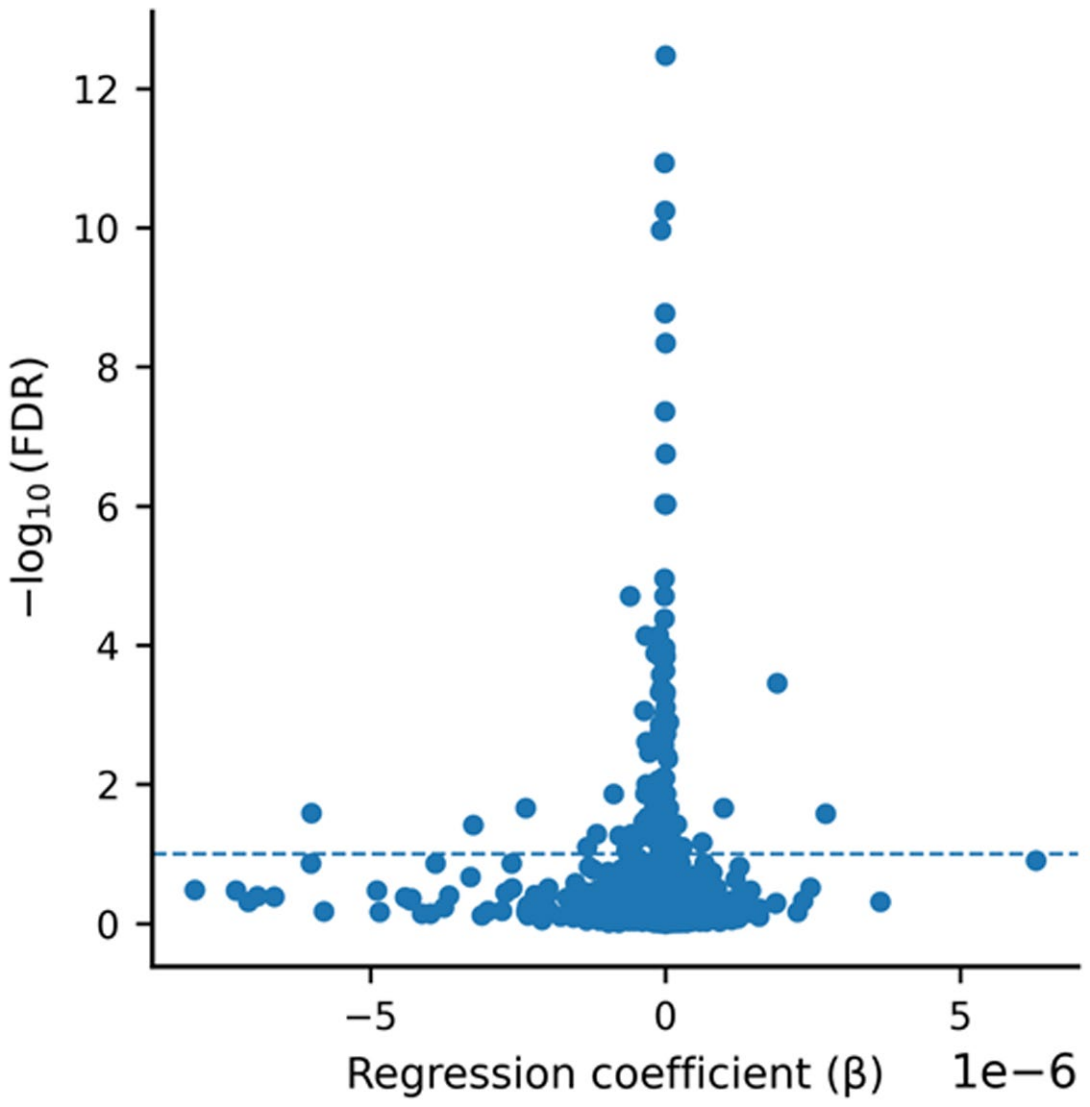
Volcano plot of regression-associated features. Each point represents a feature tested in regression analysis. The x-axis shows the regression coefficient (*β*), and the y-axis shows statistical significance expressed as -log10 (FDR). The dashed horizontal line indicates the false discovery rate threshold (*q* < 0.1).

In total, 6,234 proteins were identified, of which 137 were significantly associated with LM II_recog performance after FDR correction (*q* < 0.1). Among these, 38 proteins were upregulated and 99 were downregulated. The complete list of significant proteins, including UniProt IDs, fold changes, and adjusted *p*-values, is provided in Supplementary Table 1. To provide a more intuitive visualization of the regression results, we generated a volcano plot displaying effect sizes (*β* coefficients) against statistical significance (Figure 2). This representation highlights both the magnitude and direction of associations, as well as the features meeting the predefined FDR threshold (*q* < 0.1), allowing clearer identification of the most strongly associated proteins. Among the top ten proteins identified, nine (THTPA, PLOD1, MFN2, NGFR, PPP2R5E, LRRC59, PPP6R3, CHP1, and ITGAV) showed reduced levels (↓) in participants w ith higher cognition, whereas C4A/C4B exhibited an elevated level (↑) (Table 3). Notably, six of the top ten candidate proteins - PLOD1, MFN2, NGFR, PPP2R5E, C4A/C4B, and ITGAV - have established links to Alzheimer’s disease and/or cognitive function, highlighting their potential as biomarkers of cognitive impairment (Table 3). Out of these six, C4A/C4B only showed a positive correlation with the people with higher cognition.

**Table 3:** Biological information for the top 10 proteins.

Symbol	Regulation	Entrez gene name	GenPept/UniProt/Swiss-Prot accession	Location	Type(s)	Biological functions related to AD and cognition	References
THTPA	↓	Thiamine triphosphatase	Q9BU02	Cytoplasm	Phosphatase	-	
PLOD1	↓	Procollagen-lysine, 2- Oxoglutarate 5- Dioxygenase 1	Q02809	Cytoplasm	Enzyme	PLOD1 is a blood biomarker for fast progressing AD	Chong et al. (2013)
MFN2	↓	Mitofusin 2	O95140	Cytoplasm	Enzyme	Plays a role in mitochondrial dynamics and AD pathogenesis Improves tau pathology, neuro inflammation and cognitive function Controls the production of Aβ	Sita et al. (2020), Wang et al. (2021), and Filadi et al. (2018)
NGFR	↓	Nerve growth factor receptor	P08138-2	Plasma Membrane	Transmembrane receptor	Regulates astroglia signaling in AD pathogenesis Aβ peptides bind to NGFR Genetic risk factor for AD and amyloid deposition	Siddiqui et al. (2023), Bruno et al. (2023), and He et al. (2022)
PPP2R5E	↓	Protein phosphatase 2 Regulatory subunit B’epsilon	Q16537	Cytoplasm	Phosphatase	Decreased expression of mRNA was reported in AD APOE interacts with PPP2R5E, implicates for AD *PPP2R5E* gene is linked tobrain development & learning disorders	Sontag and Sontag (2014), Theendakara et al. (2017), and Musumeci et al. (2025)
LRRC59	↓	Leucine rich repeat containing 59	Q96AG4	Cytoplasm	Other	-	
PPP6R3	↓	Protein phosphatase 6 Regulatory subunit 3	Q5H9R7	Cytoplasm	Other	-	
CHP1	↓	Calcineurin like EF-hand protein 1	Q99653	Nucleus	Transcription regulator	-	
C4A/C4B	↑	--	P0C0L4	Other	Other	C4A is associated with MCI C4A, C4B are significantly associated with the tau phosphorylation to total tau in AD	McCarthy et al. (2019) and Panitch et al. (2021)
ITGAV	↓	Integrin subunit alpha V	P06756	Plasma membrane	Transmembrane receptor	Associated with a lower risk of dementia and cognitive impairment	Tanaka et al. (2020)

↓ -protein is down regulated in people with higher LM II Recognition; ↑ --protein is up regulated in people with higher LM II Recognition.

### 3.3 Pathway and network analysis by ingenuity pathway analysis

**Figure 3:**
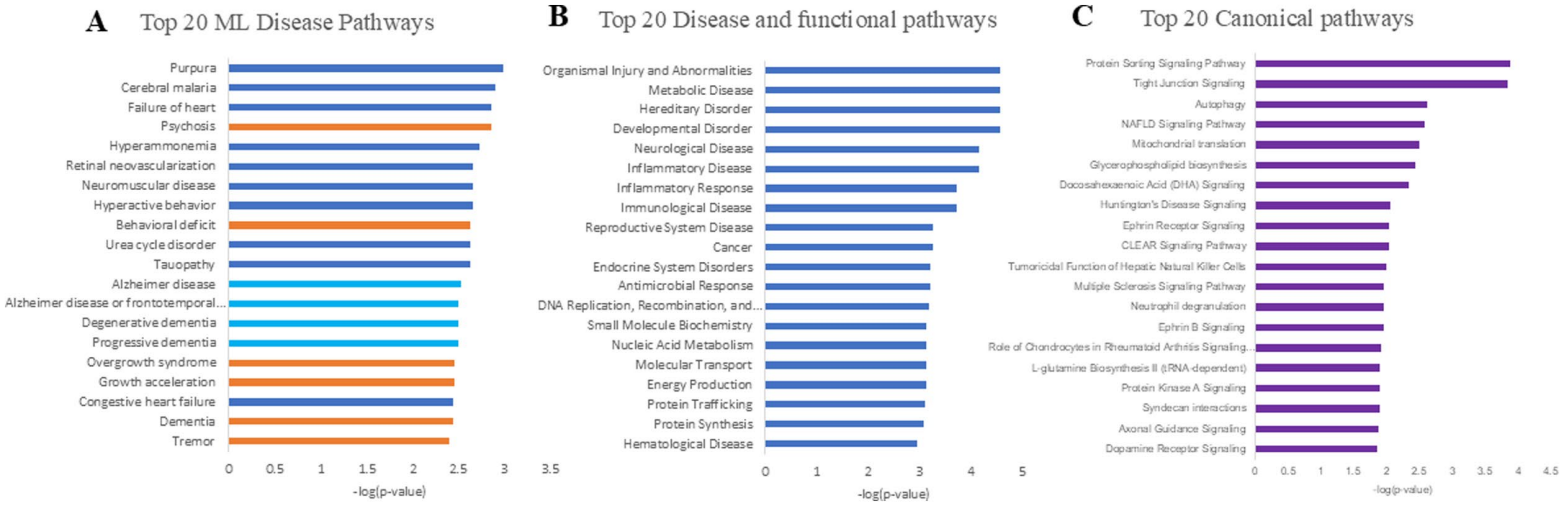
Pathways identified by IPA analysis **(A)**. Top 20 machine learning disease pathways from IPA. The most important pathways related to the neurological diseases are highlighted with orange color; **(B)** Top 20 Disease and functional pathways from IPA; **(C)** Top 20 canonical pathways from IPA.

**Figure 4:**
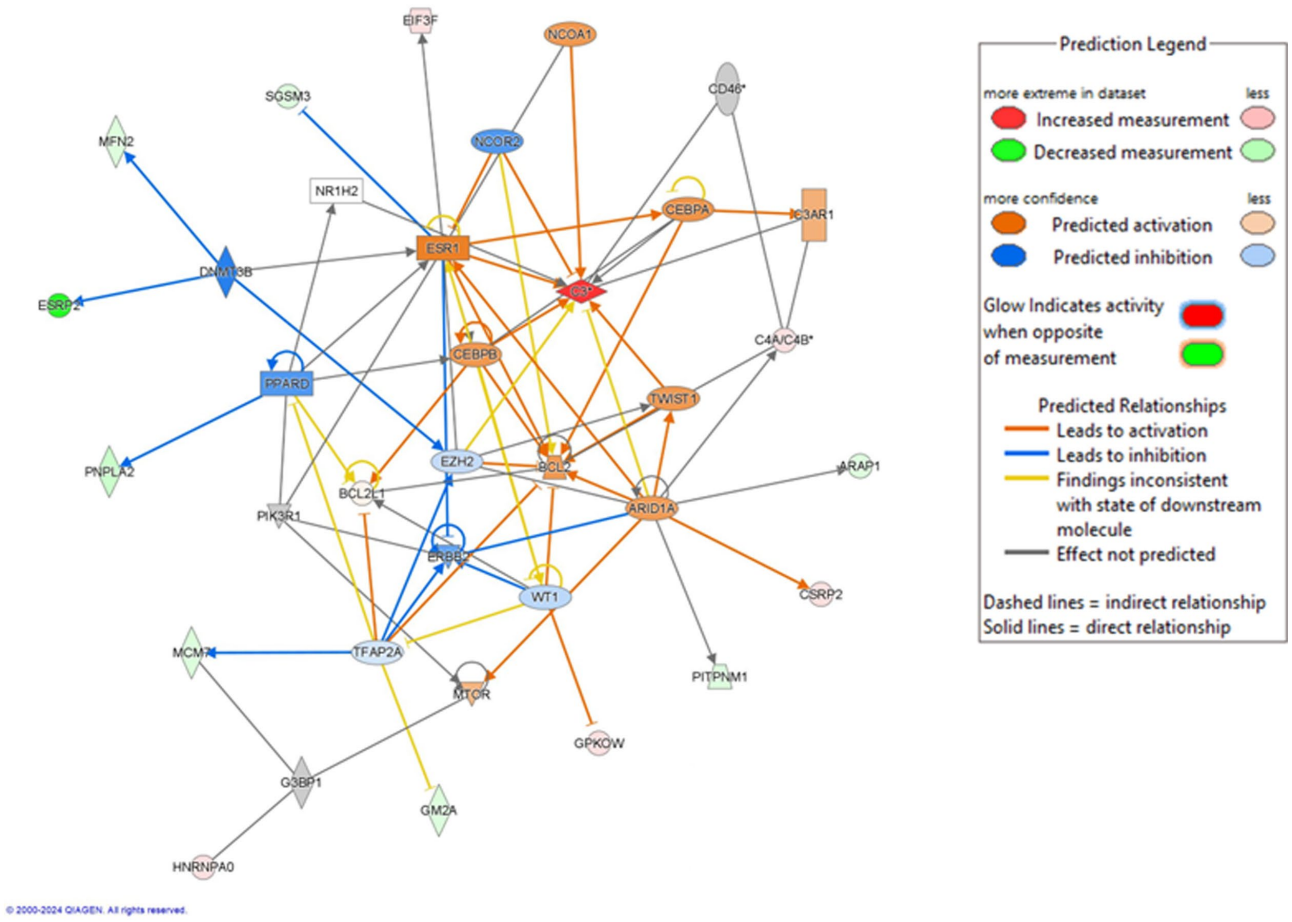
Protein network (IPA): IPA analysis of the differentially abundant proteins. Cell cycle, HNRNPAO cellular development, cellular growth and proliferati on network. 2000–2004 QIAGEN. Al rights reserved. Red color indicates increased abundance, blue color denotes decreased abundance. Green color indicates the disease processes related to the proteins (adjusted *p* < 0.1).

Ingenuity Pathway Analysis was applied to the 137 proteins associated with LM II_recog scores, using the Ingenuity Knowledge Base as a reference set that integrates published data and public protein–protein interaction databases (Krämer et al., 2014; Pane et al., 2020). Proteins were mapped to the Machine Learning (ML) disease pathways (Figure 3A), Disease and functional pathways (Figure 3B), Canonical pathways (Figure 3C), and significant molecular networks (Figure 4). Statistical significance was determined using Fisher’s exact test, and pathways were ranked by network score.

Among the top 20 ML disease pathways, seven were directly related to cognition, including Alzheimer’s disease, frontotemporal dementia, degenerative dementia, progressive dementia, psychosis, behavioral deficits, and general dementia (Figure 3A). These results highlight the role of olfactory processes in cognitive function, as reflected by molecular changes in the olfactory mucosa. In the Disease and Functional pathway analysis, four top-ranked categories - neurological disease, inflammatory disease, immunological disease, and inflammatory response - were significantly associated with LM II_recog performance (Figure 3B), consistent with the contribution of neuroinflammation to cognitive decline in aging. Canonical pathway analysis indicates that several metabolic and signaling pathways such as autophagy, CLEAR signaling pathways, multiple sclerosis signaling, ephrin receptor signaling, ephrin B signaling and L-glutamate biosynthesis, are associated with the cognition (Figure 3C). These associations observed in the olfactory mucosa may indicate broader metabolic or neurotransmitter-related vulnerabilities contributing to cognitive decline. Finally, network analysis revealed a principal molecular network (Figure 4) involving proteins linked to cell cycle regulation, cellular development, and proliferation, underscoring broader biological processes that may influence memory-related pathways.

## 4 Discussion

This study demonstrates that olfactory mucosa proteomics can reveal molecular signatures associated with LM II_recog performance in elderly African American adults without diagnosed neurological disease. Six proteins - PLOD1, MFN2, NGFR, PPP2R5E, C4A/C4B, and ITGAV - previously implicated in AD or cognition, were significantly associated with memory scores, highlighting mitochondrial and synaptic pathways, tau regulation, neurotrophin signaling, and complement-mediated neuroinflammation. Identifying these proteins in OM, a minimally invasive and accessible tissue, underscores its potential as a surrogate site for biomarker discovery in aging and dementia. The identification of 6,234 proteins in nasal exfoliate samples is higher than yields reported in several prior studies of olfactory and nasal tissues; however, this range is consistent with advances in high-resolution mass spectrometry and sample processing workflows. To ensure robustness of downstream analyses, proteins were filtered based on their detection frequency, requiring presence in at least 70% of samples within each experimental group. This resulted in a final dataset of 4,501 proteins that were consistently quantified across both groups. This filtering step was applied to reduce the influence of proteins with excessive missing values, which may arise due to stochastic sampling in mass spectrometry-based proteomics and can introduce bias during imputation. By retaining proteins with sufficient quantitative coverage, we aimed to improve the reliability and interpretability of downstream statistical analyses. For comparison, Hwang et al. performed LC–MS/MS–based proteomic analysis of human olfactory epithelial tissue and identified 3,731 proteins under stringent false discovery rate thresholds (<1%) (Hwang et al., 2018). Similarly, a study of olfactory cleft mucus by Soler et al. (2021) reported 2,514 proteins across subjects, while proteomic profiling of nasal polyp tissue using 2D-DIGE and MALDI-TOF approaches identified approximately 1,963 proteins (Farajzadeh Deroee et al., 2009).

The candidate proteins converge on biological mechanisms central to AD. PLOD1 has been proposed as a biomarker for fast-progressing AD (Chong et al., 2013). PLOD1 expression is significantly upregulated in fast-progressor AD patients and shows a negative association with LM II_recog performance, indicating that higher PLOD1 levels correspond to poorer cognitive outcomes. However, this finding is derived from peripheral blood leukocytes rather than OM tissue and may not directly reflect tissue-specific protein regulation within the central nervous system or olfactory mucosa. MFN2 regulates mitochondrial dynamics and has been linked to amyloid and tau pathology, neuroinflammation, and cognitive function (Chong et al., 2013; Filadi et al., 2018; Sita et al., 2020; Wang et al., 2021). MFN2 expression is significantly reduced in the brain tissue of patients with Alzheimer’s disease and in AD mouse models. However, in our dataset, MFN2 protein levels were lower in individuals with higher cognition, which may reflect a compensatory response occurring during early or preclinical disease stages. NGFR (p75NTR) modulates neurotrophin signaling, influencing neuronal survival and death (Bruno et al., 2023; He et al., 2022). NGFR is typically viewed as neuroprotective in the central nervous system and has been associated with larger hippocampal volume and enhanced neuronal survival. However, in our dataset, NGFR levels showed a negative association with LM II_recog, which is opposite to expectations based on previous brain studies. Taken together, our findings suggest that NGFR’s inverse association with memory performance likely reflects a stress-responsive, regenerative phenotype in the olfactory mucosa, which operates through pathways distinct from the NGFR-mediated pro-survival signaling typically described in hippocampal neurobiology. PPP2R5E, a regulatory subunit of PP2A, controls tau dephosphorylation, is repressed by ApoE4, and shows reduced expression in AD hippocampus, contributing to tau pathology (Sontag et al., 2004; Sontag and Sontag, 2014; Theendakara et al., 2017). The hippocampus plays a central role in LM II_recog performance, and our findings show that PPP2R5E is negatively correlated with LM II_recog. This inverse association suggests that higher PPP2R5E levels may reflect molecular changes linked to reduced hippocampal-dependent memory function. Complement proteins C4A/C4B are associated with tau phosphorylation and cognitive deficits in AD and schizophrenia (Jun et al., 2022; Panitch et al., 2021). Therefore, the positive correlation with LM II_recog suggests that higher levels of C4A/C4B may reflect a compensatory response during the early stages of cognitive decline. Such compensatory activation of the complement pathway has been described in preclinical phases of neurodegenerative diseases, where immune signaling temporarily increases before overt cognitive deterioration becomes evident. ITGAV has been linked to reduced dementia risk (Tanaka et al., 2020). However, its negative association with LM II_recog in our dataset suggests that lower ITGAV levels may signal early vulnerability in cognitive pathways before clinically detectable decline emerges. In addition, novel candidates (e.g., THTPA, LRRC59, PPP6R3, CHP1) expand the scope of olfactory proteomics in cognitive research. THTPA, an enzyme in thiamine metabolism and thiamine deficiency and disrupted thiamine-dependent pathways have long been implicated in impaired neuronal energy metabolism and AD progression (Gibson et al., 2016; Pan et al., 2015). LRRC59, a nuclear envelope and ER-associated protein involved in mRNA translation and protein trafficking, has not previously been directly linked to AD, but its functional role in proteostasis and ER stress aligns with molecular disturbances observed in AD brains (Hetz and Saxena, 2017). PPP6R3, a regulatory subunit of protein phosphatase 6 (PP6), participates in cell cycle control and inflammatory signaling; given that dysregulated phosphatase activity is a known driver of tau hyperphosphorylation in AD (Sontag, J. and Sontag 2014), PPP6R3 represents a plausible regulator of tau pathology and neuroinflammation. Finally, CHP1, a calcium-binding regulator of Na^+^/H^+^ exchangers, is critical for neuronal excitability and survival; mutations in CHP1 cause neurodegeneration (Mendoza-Ferreira et al., 2018), and its role in calcium signaling intersects with pathways disrupted in AD (Berridge, 2011). THTPA, LRRC59, PPP6R3, and CHP1 were negatively correlated with LM II_recog scores, suggesting that reduced levels of these proteins may play a putative role in cognitive function and could reflect early molecular vulnerability associated with lower memory performance. OM is a peripheral, dynamic tissue influenced by epithelial turnover, immune activity, and environmental exposure, and therefore may not directly mirror protein regulation observed in brain tissue. Instead, OM-derived protein levels may reflect compensatory responses, peripheral adaptations, or early interface-level processes at the nasal–brain boundary. Together, these findings highlight both established and emerging molecular connections between olfactory proteomics and cognitive impairment, broadening the scope of candidate biomarkers and suggesting mechanistic links worthy of future validation.

Pathway analyses reinforce these molecular associations. ML disease pathways enriched for AD, dementia syndromes, and behavioral deficits support the alignment between OM proteomics and cognitive performance. Disease and Functional analyses emphasized inflammation- and immunity-related pathways, consistent with reports that inflammatory proteins are enriched in olfactory mucus of older adults (Yoshikawa et al., 2018). Such findings align with the concept of “inflammaging,” in which chronic low-grade inflammation accelerates cognitive decline (Lepperdinger, 2011; Tuppo and Arias, 2005). Importantly, prior studies of olfactory mucus have also linked inflammatory regulators such as S100A8/A9 to amyloid plaque formation and memory decline in AD models (Gebhardt et al., 2006; Tuppo and Arias, 2005; Yoshikawa et al., 2018). Together, these results emphasize that OM proteomics capture both neurodegenerative and inflammatory processes relevant to cognition. African American populations exhibit a distinct risk profile for Alzheimer’s disease, including higher burden of vascular comorbidities, differential stress biology, and potential variation in APOE ε4–associated risk. These factors may influence underlying disease mechanisms and biomarker expression, highlighting the importance of studying molecular signatures within this population (Logue et al., 2023). In our cohort APOE4 prevalence is 43.9%—roughly twice that observed in the general population—underscores the heightened genetic vulnerability of the study cohort (Farrer et al., 1997).

Despite these promising findings, several limitations should be acknowledged. First, because the study cohort was predominantly female, it will be important to investigate proteins that are modulated by estrogen or interact with estrogen-sensitive pathways, as hormonal influences may underlie sex-specific differences in cognitive resilience and vulnerability to neurodegeneration (Arenaza-Urquijo et al., 2024; Lu et al., 2025). The significant proteins and pathways were not validated by targeted proteomic approaches, limiting immediate translational application. We acknowledge the limited generalizability of our findings to other racial and ethnic populations. Therefore, future studies are needed to validate these proteomic markers across racially and ethnically diverse cohorts.

The modest sample size also reduces statistical power and generalizability. Future research should include larger, multi-ethnic cohorts and targeted validation to confirm these candidate markers and clarify their mechanistic role in cognitive decline. The cross-sectional design precludes any causal inferences regarding the identified proteins and the progression of cognitive decline. While our cohort was recruited from a single site in the Washington, DC area and comprised individuals with subjective cognitive complaints, the high mean MMSE score (28.1) suggests that many participants maintain objectively normal cognitive function. We observed a notable ceiling effect on the FCSRT (mean 46.93, range 38–48), which is common in non-demented populations and justified our use of LM II_recog – a measure with greater variance in this cohort – as the primary outcome. Furthermore, although we adjusted for APOE ε4 status in our regression models to account for its known influence on neurobiology, the findings require validation in larger, multi-ethnic, longitudinal cohorts to determine their diagnostic and predictive utility. A further consideration involves the cellular composition of the nasal exfoliates. While our sampling was anatomically targeted to the upper nasal cavity by trained otolaryngologists to maximize the yield of olfactory mucosa (OM), these samples inherently consist of a heterogeneous mixture of olfactory receptor neurons, sustentacular cells, and respiratory epithelial cells. Although we confirmed the presence of mature olfactory neurons via DIC microscopy and marker staining, the proteomic profiles likely reflect a combination of olfactory and respiratory signals. Future studies utilizing single-cell proteomics or more invasive biopsy techniques may be necessary to further isolate neuron-specific contributions to the cognitive biomarkers identified here.

## 5 Conclusion

This pilot study identifies olfactory mucosa proteomic markers and pathways associated with LM II_recog performance in elderly African Americans. Six key proteins - PLOD1, MFN2, NGFR, PPP2R5E, C4A/C4B, and ITGAV - alongside inflammation- and neurodegeneration-related pathways highlight the OM as a practical and promising source for biomarker discovery. These findings provide novel insights into population-specific mechanisms of cognitive decline and lay the foundation for future studies to validate these proteins as diagnostic or therapeutic targets in aging and dementia.

## Data Availability

The data that supports the findings are not publicly available due to privacy and/or ethical reasons. Further inquiries should be directed to the corresponding author/s.
