# Supplementary figures and images for: Proteomic profiling of olfactory exfoliates from people with subjective cognitive complaints reveal networks of olfactory biomarkers of cognitive performance
Source: Front Aging Neurosci. 2026 May 21;18:1781518. doi: 10.3389/fnagi.2026.1781518 (PMC13235274; doi:10.3389/fnagi.2026.1781518)

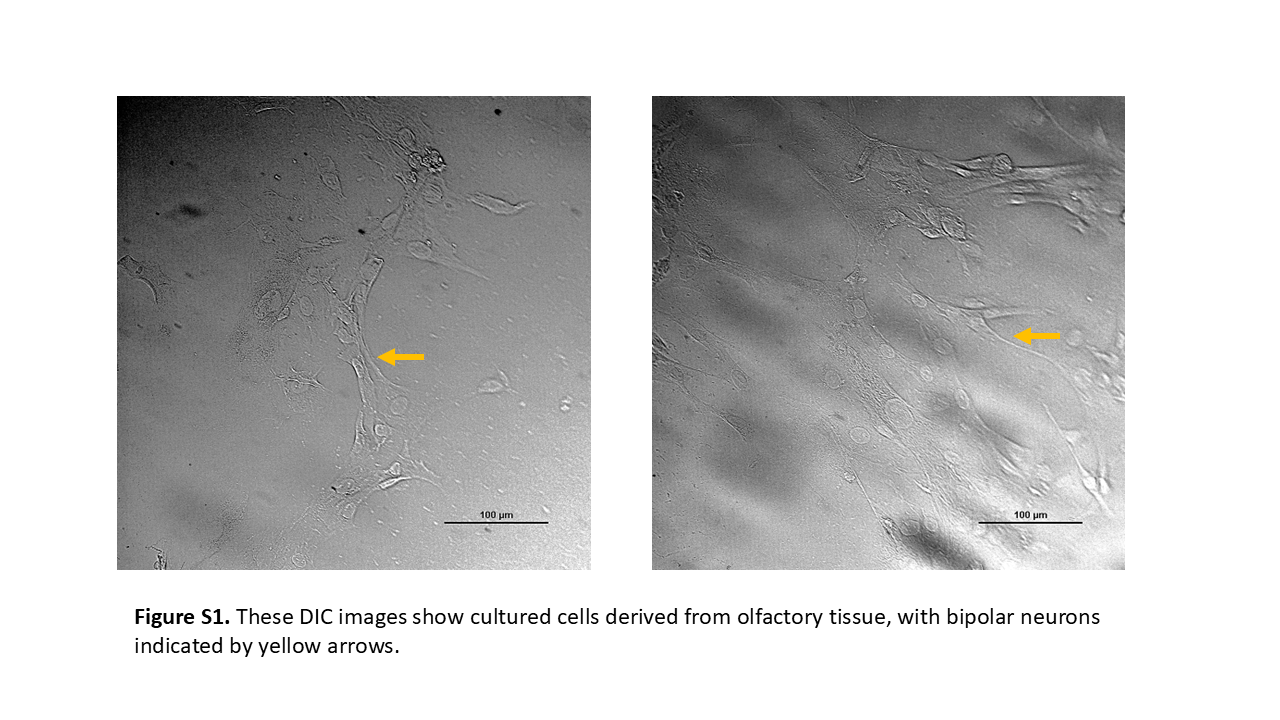

Supplement: Supplementary file 1 [file Image_1.TIF]

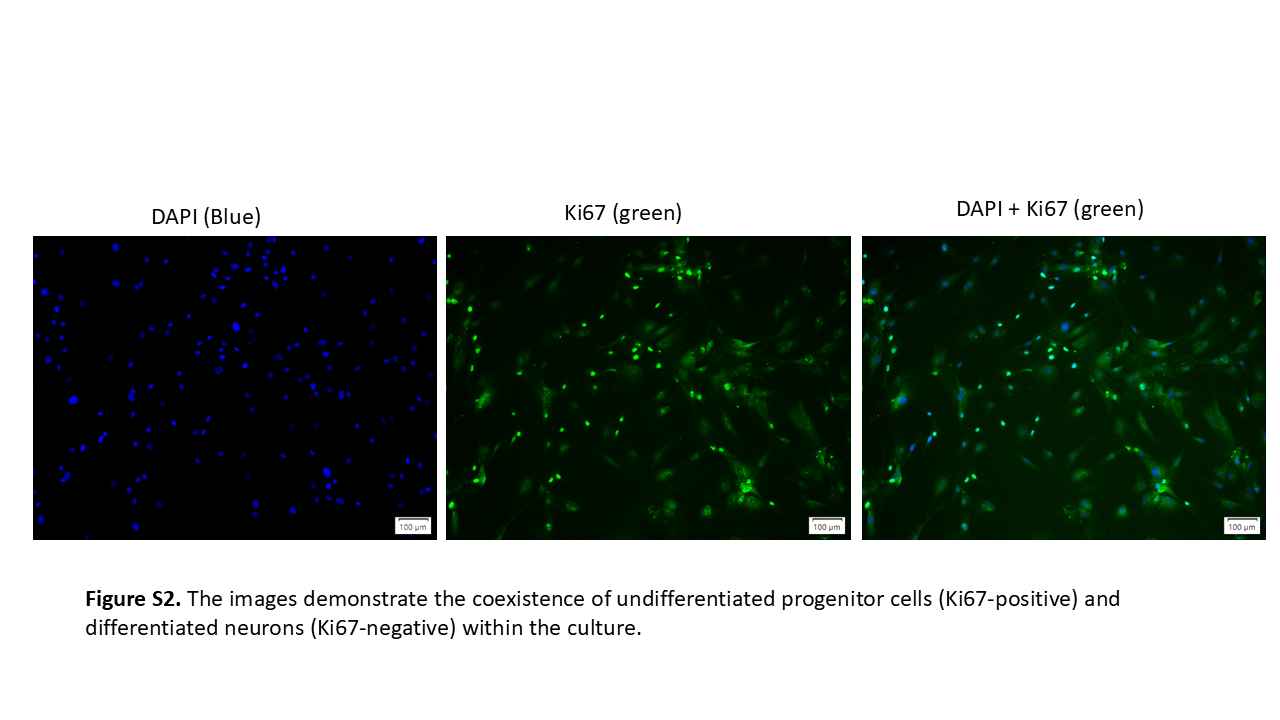

Supplement: Supplementary file 2 [file Image_2.TIF]

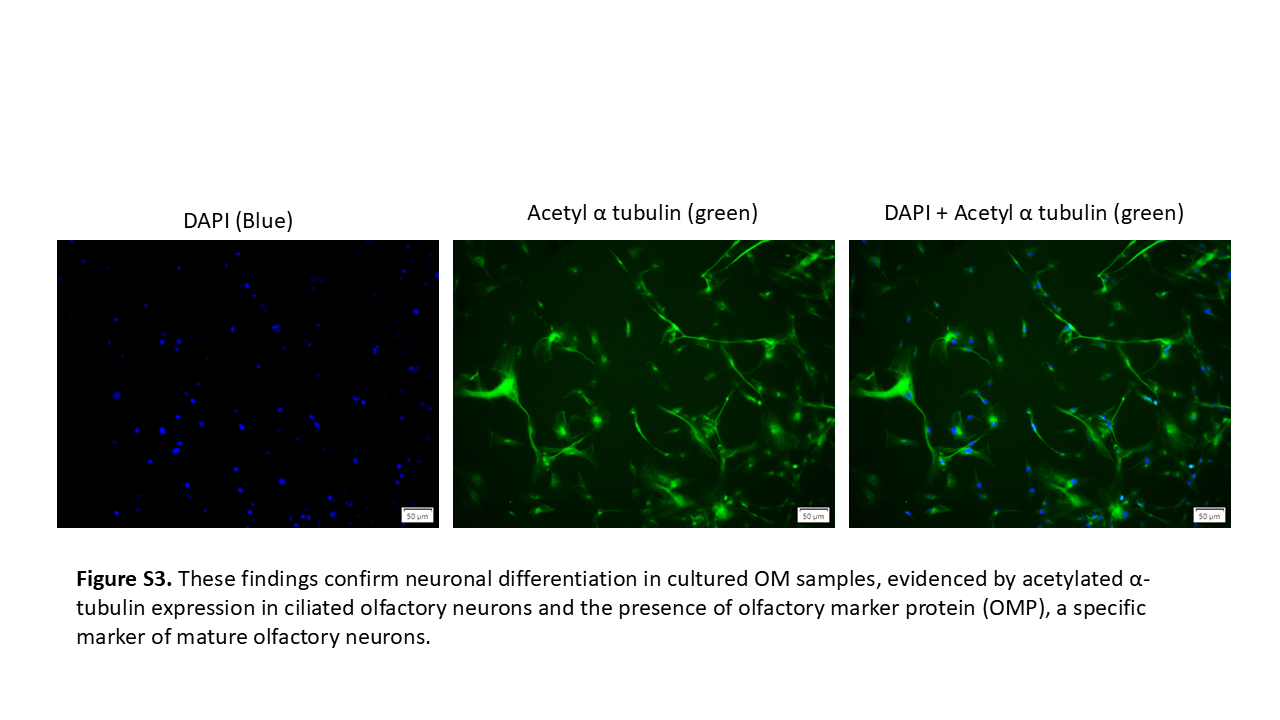

Supplement: Supplementary file 3 [file Image_3.TIF]

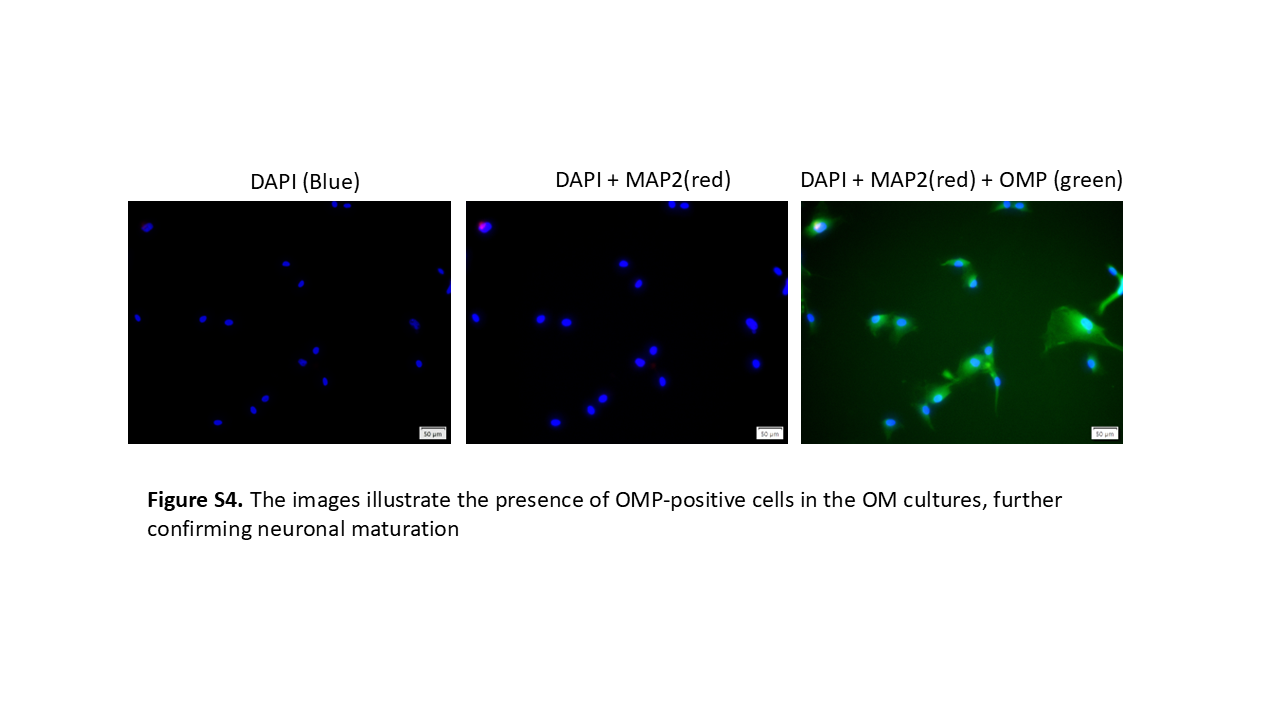

Supplement: Supplementary file 4 [file Image_4.TIF]
