# Supplementary material for: Proteomic profiling of olfactory exfoliates from people with subjective cognitive complaints reveal networks of olfactory biomarkers of cognitive performance
Source: Front Aging Neurosci. 2026 May 21;18:1781518. doi: 10.3389/fnagi.2026.1781518 (PMC13235274; doi:10.3389/fnagi.2026.1781518)
Supplement: Supplementary file 5 [file Table_1.DOCX]

**Table 1. Demographic features and APOE genotype frequencies of study participants.**

| **Demography** | **N= 108 (%)** |
| --- | --- |
| **Gender** |  |
| Male | 32 (29.63) |
| Female | 76 (70.37) |
| **Race** |  |
| Asian | 1 (0.93) |
| Biracial | 1 (0.93) |
| Black | 94 (87.04) |
| Other | 1 (0.93) |
| white | 11 (10.19) |
| **Marital Status** |  |
| Divorced | 21 (19.44) |
| Married | 25 (23.15) |
| Married twice | 2 (1.85) |
| Never Married | 44 (40.74) |
| Separated | 1 (0.93) |
| Widowed | 15 (13.89) |
| **Employment Status** |  |
| Employed | 17 (15.74) |
| Retired | 57 (52.78) |
| Self employed | 5 (4.63) |
| Unemployed | 26 (24.07) |
| Unemployed (disability) | 3 (2.78) |
| **ApoE genotypes** |  |
| Non E4 carrier (E2/E2, E2/E3, E3/E3) | 61 (56.48) |
| Heterozygous E4 (E2/E4, E3/E4) | 43 (39.82) |
| E4 Homozygous (E4/E4) | 4 (3.7) |
| **Clinical characteristics of the study participants** | **Mean (SD)** |
| Age | 66.65 (7.71) |
| Education | 14.29 (3.42) |
| MMSE | 28.13 (2.053) |
| Story B | 8.35 (4.11) |
| DSST | 42.41 (15.93) |
| LM II_Recog | 20.06 (3.56) |
| FCSRT | 46.93 (1.96) |

**MMSE:** Mini Mental State Examination; **DSST:** Digit-Symbol-Substitution Test;

**LM II:** Logical Memory II; **FCSRT:** Free and Cued Selective Reminding Test

**Table 2. Correlation matrix of clinical variables.**

|  | **LM II_R** | **MMSE** | **FCSRT** | **DSST** | **Story B** | **ADCS-PACC** | **TDI** |
| --- | --- | --- | --- | --- | --- | --- | --- |
| **LM II_R** | 1.0000 |  |  |  |  |  |  |
| **MMSE** | 0.4380*** | 1.0000 |  |  |  |  |  |
| **FCSRT** | 0.2838** | 0.2882** | 1.0000 |  |  |  |  |
| **DSST** | 0.4641*** | 0.3461** | 0.1501 | 1.0000 |  |  |  |
| **Story B** | 0.6642*** | 0.4962*** | 0.2689* | 0.3995*** | 1.0000 |  |  |
| **ADCS-PACC** | 0.5397*** | 0.4669*** | 0.2335* | 0.7995*** | 0.6273*** | 1.0000 |  |
| **TDI** | 0.3541*** | 0.2256* | 0.1866 | 0.1238 | 0.1850 | 0.1417 | 1.000 |

*** p<0.001

** p<0.01

* p<0.05

LM-R, logical memory II recognition; MMSE, Mini-Mental Status Examination; FCSRT, Free and Cued Selective Reminding Test; ADCS-PACC, DSST, Digit-Symbol-Substitution Test; TDI, Odor Threshold, Discrimination and Identification Index.
